# Editorial: Machine Learning in Biomolecular Simulations

**DOI:** 10.3389/fmolb.2019.00076

**Published:** 2019-08-29

**Authors:** Gennady Verkhivker, Vojtech Spiwok, Francesco Luigi Gervasio

**Affiliations:** ^1^Graduate Program in Computational and Data Sciences, Schmid College of Science and Technology, Chapman University, Orange, CA, United States; ^2^Department of Biomedical and Pharmaceutical Sciences, Chapman University School of Pharmacy, Irvine, CA, United States; ^3^Department of Biochemistry and Microbiology, University of Chemistry and Technology, Prague, Czechia; ^4^Protein Dynamics Research Group, Chemistry Department, University College London, London, United Kingdom

**Keywords:** machine learning, molecular modeling, molecular dynamics computer simulation, intrinsically disordered protein, sampling enhancement, non-linear dimension reduction

Interest in machine learning is growing in all fields of science, industry, and business. This interest was not primarily initiated by new theoretical findings. Interestingly, the theoretical basis of the majority of machine learning techniques, such as artificial neural networks, decision trees, or kernel methods, have been known for a relatively long time. Instead, there are other effects that triggered the recent boom of machine learning.

First, machine learning needs data to learn on. Huge data sets from Internet, Internet of Things, social networks, phones, wearable devices, and other sources are now available. Such datasets were not available a decade ago. Second, the recent wave of machine learning benefits from hardware advances, in particular from computing on graphical processing units and specialized hardware.

Biomolecular modeling and simulations are an ideal field for the application of machine learning approaches in the spirit of the recent boom of machine learning. Biomolecular simulations produce large amounts of data in the form of trajectories that can be used to train machine learning algorithms. At the same time, vast amounts of genomic data were critical in allowing AlphaFold in leading the field of *de novo* protein prediction in the most recent CASP protein prediction round. Moreover, GPUs are routinely used in biomolecular simulations for more than a decade to offload critical parts of calculation.

This Research Topic collects eight innovative works showcasing the application of machine learning in biomolecular simulations and related fields. It demonstrates major machine learning approaches such as artificial neural networks, random forests, and non-linear dimensionality reduction methods. These techniques are applied in analysis of trajectories, acceleration of biomolecular simulations, parametrization of force fields, and other tasks.

Helfrecht et al. present an alternative to classical definitions of structural motifs in proteins. Classical definitions of secondary and super-secondary structures are based on intuitive criteria, such as hydrogen bonds, dihedral angles, and others and have been widely used. However, they experience problems with borderline and partially disordered structures. This article presents an alternative based on machine learning, namely on Probabilistic Analysis of Molecular Motifs algorithm previously developed in the group.

The article from Trapl et al. presents a program Anncolvar. This tool makes it possible to approximate a collective variable using a simple neural network. The choice of optimal collective variables is crucial to the convergence of enhanced algorithms based on them. Anncolvar is shown to be very useful for collective variables that cannot be explicitly calculated on-the-fly or computationally expensive collective variables.

Wang et al. used classical as well as by unsupervised and supervised machine learning methods (principal component analysis, random forest) to analyze protein dynamics. They analyzed trajectories of an enzyme linked to antibiotic resistance β-lactamase, simulated in multiple conformational states.

Intrinsically disordered proteins (IDPs) are a hot topic given that about 10% of all proteins are disordered, and about 40% of eukaryotic proteins have at least one long disordered loop. It has been shown that proteins can have a function despite not having a stable conformation. This brings a new challenge in analysis of dynamics. Grazioli et al. use machine learning and network models on simulation trajectories of amyloid beta in its wild type and its medicinally relevant mutant. They show that machine learning analysis can explain the difference between protein variants. This was not possible by conventional trajectory analysis methods.

There is a growing number of works indicating that molecular mechanics potentials (force fields) developed for compactly folded proteins may fail in modeling of unfolded proteins and especially IDPs. This fact motivated Demerdash et al. to optimize force field for IDPs on the basis of data from small-angle X-ray/neutron scattering. This was done by iterative rounds of molecular dynamics simulations and comparison with experimental data. This approach was demonstrated on three IDPs.

The article of Agajanian et al. drives us more into the bioinformatics area. Recent applications of next-generation sequencing makes it possible to identify the role of mutations associated with cancer. The authors integrated multiple machine learning approaches to classify mutations an the basis of nucleotide sequence. The approach is further illustrated on biomolecular simulations of cancer associated protein kinases.

Tribello and Gasparotto use unsupervised machine learning methods to analyse simulation trajectories. Trajectory of the C-terminal fragment of the immunoglobulin binding domain B1 of protein G of *Streptococcus* was used as a model trajectory and analyzed by a range of mostly non-linear dimensionality reduction methods, namely principal component analysis, distance matching, Laplacian eigenmaps, Isomap, tSNE, and sketchmap. These methods are illustrated together with clustering methods. The article provides an overview of these methods and their advantages and disadvantages are discussed.

Kinetics of drug unbinding is recently becoming equivalently or even more important than binding thermodynamics in drug design as a parameter distinguishing between good and bad compounds. The article of Kokh et al. addresses this problem by machine learning. There are several trajectories of spontaneous drug binding available in literature. Drug unbinding is several orders of magnitude slower and today cannot be simulated without enhanced sampling. The authors analyzed a series of trajectories from enhanced sampling method Random Accelerated Molecular Dynamics, in particular its variant designed for simulation of drug unbinding kinetics. The approach has been tested on a series of heat shock protein 90 ligands differing by four orders of magnitude in their unbinding rates. Excellent agreement with experiment was obtained for most classes of compounds.

We believe that the papers included in this Research Topic demonstrate the great potential of machine learning in all fields pertaining biomolecular modeling and simulations, including in improving the accuracy of the models, in the analysis of molecular simulations and in providing effective variables to enhance the sampling. With this Research Topic *Frontiers in Molecular Biosciences* aspires to become a key forum for publishing of approaches combining machine learning with biomolecular simulations and further promote this multidisciplinary field.

## Author Contributions

All authors listed have made a substantial, direct and intellectual contribution to the work, and approved it for publication.

### Conflict of Interest Statement

The authors declare that the research was conducted in the absence of any commercial or financial relationships that could be construed as a potential conflict of interest.

